# Disease-Dependent Antiapoptotic Effects of Cannabidiol for Keratinocytes Observed upon UV Irradiation

**DOI:** 10.3390/ijms22189956

**Published:** 2021-09-15

**Authors:** Piotr Wójcik, Agnieszka Gęgotek, Neven Žarković, Elżbieta Skrzydlewska

**Affiliations:** 1 Department of Analytical Chemistry, Medical University of Bialystok, 15-222 Bialystok, Poland; piotr.wojcik@umb.edu.pl (P.W.); agnieszka.gegotek@umb.edu.pl (A.G.); 2 LabOS, Rudjer Boskovic Institute, 10000 Zagreb, Croatia; Neven.Zarkovic@irb.hr

**Keywords:** CBD, skin inflammation, UVB, keratinocytes, apoptosis

## Abstract

Although apoptosis of keratinocytes has been relatively well studied, there is a lack of information comparing potentially proapoptotic treatments for healthy and diseased skin cells. Psoriasis is a chronic autoimmune-mediated skin disease manifested by patches of hyperproliferative keratinocytes that do not undergo apoptosis. UVB phototherapy is commonly used to treat psoriasis, although this has undesirable side effects, and is often combined with anti-inflammatory compounds. The aim of this study was to analyze if cannabidiol (CBD), a phytocannabinoid that has anti-inflammatory and antioxidant properties, may modify the proapoptotic effects of UVB irradiation in vitro by influencing apoptotic signaling pathways in donor psoriatic and healthy human keratinocytes obtained from the skin of five volunteers in each group. While CBD alone did not have any major effects on keratinocytes, the UVB treatment activated the extrinsic apoptotic pathway, with enhanced caspase 8 expression in both healthy and psoriatic keratinocytes. However, endoplasmic reticulum (ER) stress, characterized by increased expression of caspase 2, was observed in psoriatic cells after UVB irradiation. Furthermore, decreased p-AKT expression combined with increased 15-d-PGJ_2_ level and p-p38 expression was observed in psoriatic keratinocytes, which may promote both apoptosis and necrosis. Application of CBD partially attenuated these effects of UVB irradiation both in healthy and psoriatic keratinocytes, reducing the levels of 15-d-PGJ_2_, p-p38 and caspase 8 while increasing Bcl2 expression. However, CBD increased p-AKT only in UVB-treated healthy cells. Therefore, the reduction of apoptotic signaling pathways by CBD, observed mainly in healthy keratinocytes, suggests the need for further research into the possible beneficial effects of CBD.

## 1. Introduction

Skin diseases are usually neglected among numerous age- and stress-associated disorders. However, autoimmune and inflammatory diseases that affect the skin by pathological interactions between keratinocytes and immune cells can happen almost at any age and often progress with aging. Among these diseases, the best known is psoriasis, which is caused by pathological interactions between skin-infiltrating leukocytes (especially lymphocytes) and keratinocytes [1], by activating immune cells and further increasing their migration into the skin. As a consequence, psoriatic plaques develop as inflammatory foci and oxidative stress, caused by the excessive production of reactive oxygen species (ROS), mainly by activated immune cells [2,3].

The main effect of this chronic autoimmune disorder is persistent oxidative stress and disturbed apoptosis of hyperproliferating epidermal cells. Apoptosis is a process that leads to the elimination of damaged or aged cells, and occasionally even those cells that have undergone malignant transformation [2,3]. This process is crucial for controlling cell growth and fate and may be triggered by activation of death receptors, DNA damage or the accumulation of abnormal proteins in the endoplasmic reticulum (ER), leading to the activation of extrinsic, intrinsic or (ER) stress-induced pathways [2,3,4]. All these pathways are characterized by specific activated caspases [5]. The exact regulatory mechanisms of apoptosis are still not fully understood, but some proteins are known to activate while others inhibit apoptosis. Among them, the balance between antiapoptotic B-cell lymphoma 2 protein (Bcl2) and proapoptotic Bcl-2-associated X protein (BAX) seems to be a most important regulator of the intrinsic pathway [6,7]. Conversely, the ER stress-induced pathway is activated by damaged and nondegraded proteins [4,8]. Finally, the extrinsic pathway is activated by proapoptotic factors that activate death receptors, especially tumor necrosis factor receptor 1 (TNFR1) [3].

As mentioned above, the lack of apoptosis is in the case of psoriasis also associated with increased cell proliferation in psoriatic patients. Although not entirely understood such “overproliferation” of psoriatic keratinocytes may be caused by the oxidative stress that leads to the breakdown of complexes between Kelch-like ECH-associated protein 1 (Keap1) and nuclear factor erythroid 2-related factor 2 (Nrf2). Once Nrf2 is activated, this can lead to activation of the mTOR pathway [9], which increases cells proliferation [10,11,12].

The high incidence of psoriasis, its uncertain pathogenesis and its tendency for progression make it an important medical and social challenge. In order to reduce the severity of psoriatic lesions, UV irradiation is often employed [13]. Interestingly this irradiation is not used to destroy the affected skin tissue itself directly; rather, it is used to exert proapoptotic effects on the lesions and eventually reduce their growth and the number of skin-infiltrating leukocytes [14]. However, UV irradiation can also cause side effects such as inflammation and damage to normal skin, consequently being a trigger factor of psoriasis. Options for integrative biomedicine treatments combining UV therapy with anti-inflammatory compounds seem attractive and aim to eliminate the negative and intensify the positive effects of UV irradiation. Cannabidiol (CBD), a phytocannabinoid that not only has anti-inflammatory properties but also is a powerful antioxidant [15,16,17], seems to be a potentially promising compound for such therapy. CBD has positive effects on psoriatic skin cells [17,18,19] and can protect healthy cells from the negative effects of UV irradiation. Furthermore, each apoptotic pathway can be modulated by CBD and by UV irradiation [20,21]. Moreover, CBD acts as an activator of the Nrf2 transcription factor, which may further induce the transcription of the antiapoptotic protein Bcl2 [22,23,24]. However, CBD may also inhibit the expression of antiapoptotic proteins, such as protein kinase B (AKT), and increase the release of cytochrome c from mitochondria, which enhances apoptosis [25].

Although caspase action and some apoptotic pathways have been relatively well studied, there is a lack of information on the effects of biotherapies on apoptosis within both healthy and diseased cells. Therefore, the aim of this study was to evaluate possible differences in the action of CBD on the apoptosis of healthy and psoriatic keratinocytes following exposure to UVB radiation.

## 2. Results

**Figure 1:**
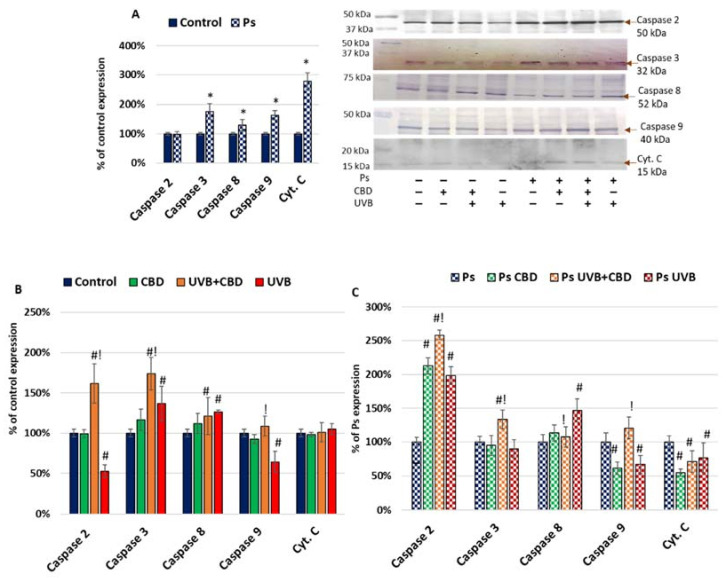
(**A**) Expression of apoptosis markers in keratinocyte cell lines from healthy subjects (Control; *n* = 5) and psoriatic patients (Ps; *n* = 5). Here, 100% corresponds to the level of protein in healthy keratinocytes untreated with UVB or CBD. The mean values ± SD are presented with statistically significant differences: * between keratinocytes from patients with psoriasis vulgaris and healthy subjects, *p* < 0.05. (**B**) Expression of apoptosis markers in keratinocyte cell lines from healthy subjects (Control; *n* = 5) without treatment and after irradiation with UVB (60 mJ/cm^2^) and/or incubation with CBD (4 µM). Here, 100% corresponds to the level of protein in healthy keratinocytes untreated with UVB or CBD. The mean values ± SD are presented with statistically significant differences: # between keratinocytes treated with CBD, UVB and UVB+CBD and nontreated, *p* < 0.05; ! between keratinocytes treated with UVB+CBD and irradiated with UVB, *p* < 0.05. (**C**) Expression of apoptosis markers in keratinocyte cell lines from psoriatic patients (Ps; *n* = 5) without treatment and after irradiation with UVB (60 mJ/cm^2^) and/or incubation with CBD (4 µM). Here, 100% corresponds to the level of protein in psoriatic keratinocytes untreated with UVB or CBD. The mean values ± SD are presented with statistically significant differences. # between keratinocytes treated with CBD, UVB and UVB+CBD and nontreated, *p* < 0.05; ! between keratinocytes treated with UVB+CBD and irradiated with UVB, *p* < 0.05.

In psoriatic cells, the expression of caspases 3, 8 and 9 (involved in intrinsic and extrinsic apoptotic pathways) was increased, suggesting that these pathways are activated under psoriasis development (Figure 1). Enhanced apoptosis in psoriatic keratinocytes was also confirmed by the increased level of cytochrome c. The UVB irradiation upregulated the expression of caspases 3 and 8 and downregulated the expression of caspase 9 in keratinocytes of healthy people. A similar effect of the UVB light was observed in the case of caspases 8 and 9 in psoriatic cells. Moreover, in psoriatic keratinocytes, UVB increased caspase 2 but slightly decreased caspase 3 expression, which was opposite to the case of healthy cells. Additionally, CBD also increased the caspase 2 level in psoriatic cells. Consequently, the combined application of both UVB irradiation and CBD increased the expression of caspases 2, 3 and 9 in keratinocytes from both healthy people and psoriatic patients. The expression of cytochrome c in healthy keratinocytes was not influenced by the applied treatments, while its reduction was observed in psoriatic cells, which initially had a much higher level of cytochrome c than the healthy cells.

**Figure 2:**
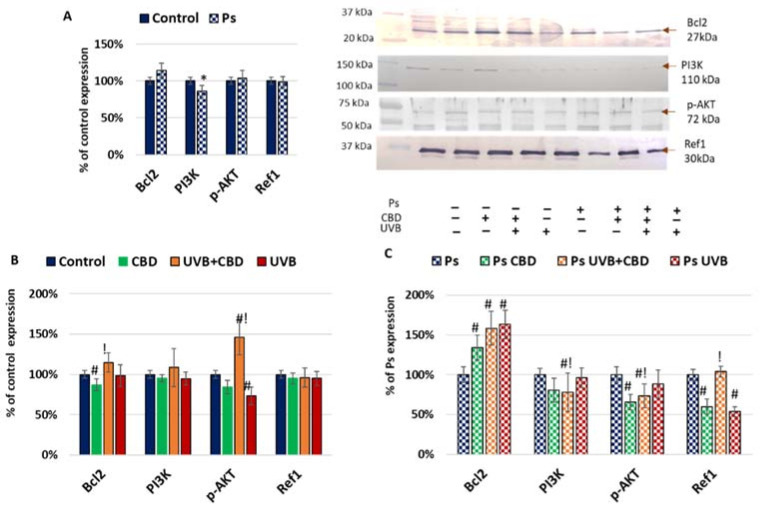
(**A**) Expression of apoptosis inhibitors in keratinocyte cell lines from healthy subjects (Control; *n* = 5) and psoriatic patients (Ps; *n* = 5). Here, 100% corresponds to the level of protein in healthy keratinocytes untreated with UVB or CBD. The mean values ± SD are presented with statistically significant differences: * between keratinocytes from patients with psoriasis vulgaris and healthy subjects, *p* < 0.05. (**B**) Expression of apoptosis inhibitors in keratinocyte cell lines from healthy subjects (Control; *n* = 5) without treatment and after irradiation with UVB (60 mJ/cm^2^) and/or incubation with CBD (4 µM). Here, 100% corresponds to the level of protein in healthy keratinocytes untreated with UVB or CBD. The mean values ± SD are presented with statistically significant differences: # between keratinocytes treated with CBD, UVB and UVB+CBD and nontreated, *p* < 0.05; ! between keratinocytes treated with UVB+CBD and irradiated with UVB, *p* < 0.05. (**C**) Expression of apoptosis inhibitors in keratinocyte cell lines from psoriatic patients (Ps; *n* = 5) without treatment and after irradiation with UVB (60 mJ/cm^2^) and/or incubation with CBD (4 µM). Here, 100% corresponds to the level of protein in keratinocytes untreated with UVB or CBD. The mean values ± SD are presented with statistically significant differences: # between keratinocytes treated with CBD, UVB and UVB+CBD and nontreated, *p* < 0.05; ! between keratinocytes treated with UVB+CBD and irradiated with UVB, *p* < 0.05.

Differences in the expression of caspases can be caused by different levels of apoptosis activators and inhibitors (Figure 2). In psoriatic keratinocytes, lower expression of phosphoinositide 3-kinase (PI3K) suggested that the antiapoptotic axis of PI3K–AKT was inhibited in these cells. On the contrary, in psoriatic keratinocytes, an increased expression of Bcl2 was noticed, which may inhibit caspase 9 activation and the release of cytochrome c from mitochondria. This may be the most important factor protecting psoriatic keratinocytes from apoptosis. Bcl2 levels in psoriatic keratinocytes were elevated by both UVB radiation and CBD treatment as well as both factors used together compared to untreated cells. On the other hand, CBD treatment reduced the level of Bcl2, while the use of both CBD and UVB increased the level of this protein in healthy cells. The Ref1 level in psoriatic keratinocytes was reduced after their treatment with UVB or CBD alone but was higher in the UVB+CBD group than in the UVB or CBD groups. However, there was no Ref1 reaction of healthy keratinocytes to the action of the factors used.

As Bcl2 acts by inhibition of generation of channels in mitochondria, which prevent the release of cytochrome c, its activation after treatment in psoriatic keratinocytes may be responsible for the lower level of cytochrome c in this group. In healthy cells, both Bcl2 and p-AKT expression were higher in the UVB+CBD group than in cells treated with CBD or UVB separately, as CBD alone decreased the Bcl2, while UVB alone decreased the p-AKT expression. p-AKT can be activated by PI3K; thus, in psoriasis, changes in PI3K and p-AKT are similar. Hence, different protective antiapoptotic mechanisms were activated in psoriatic and in healthy keratinocytes after UVB exposure.

**Figure 3:**
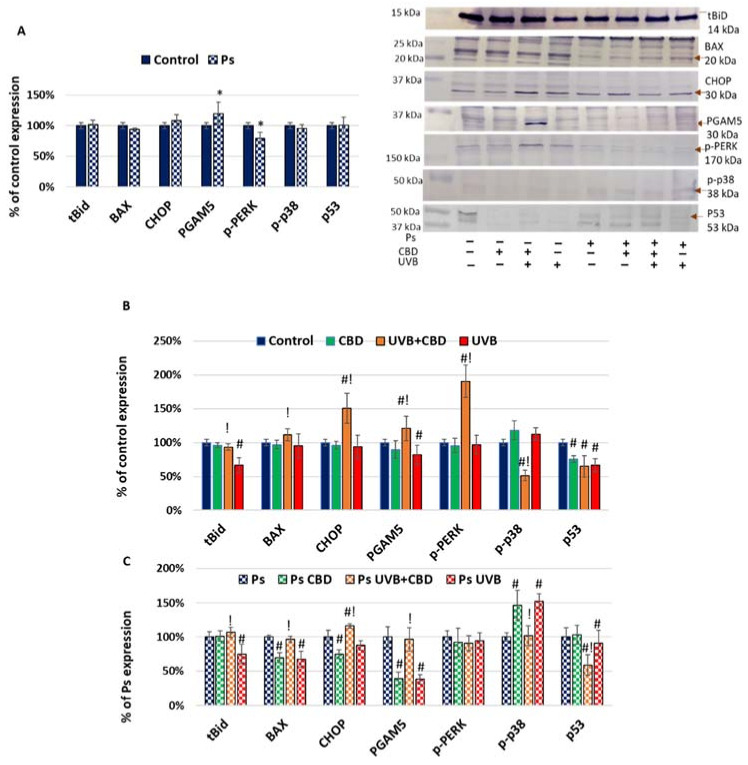
(**A**) Expression of apoptosis activators in keratinocyte cell lines from healthy subjects (Control; *n* = 5) and psoriatic patients (Ps; *n* = 5). Here, 100% corresponds to the level of protein in healthy keratinocytes untreated with UVB or CBD. The mean values ± SD are presented with statistically significant differences: * between keratinocytes from patients with psoriasis vulgaris and healthy subjects, *p* < 0.05. (**B**) Expression of apoptosis activators in keratinocyte cell lines from healthy subjects (Control; *n* = 5) without treatment and after irradiation with UVB (60 mJ/cm^2^) and/or incubation with CBD (4 µM). Here, 100% corresponds to the level of protein in healthy keratinocytes untreated with UVB or CBD. The mean values ± SD are presented with statistically significant differences: # between keratinocytes treated with CBD, UVB and UVB+CBD and nontreated, *p* < 0.05; ! between keratinocytes treated with UVB+CBD and irradiated with UVB, *p* < 0.05. (**C**) Expression of apoptosis activators in keratinocyte cell lines from psoriatic patients (Ps; *n* = 5) without treatment and after irradiation with UVB (60 mJ/cm^2^) and/or incubation with CBD (4 µM). Here, 100% corresponds to the level of protein in psoriatic keratinocytes untreated with UVB or CBD. The mean values ± SD are presented with statistically significant differences: # between keratinocytes treated with CBD, UVB and UVB+CBD and nontreated, *p* < 0.05; ! between keratinocytes treated with UVB+CBD and irradiated with UVB, *p* < 0.05.

The results obtained by the analysis of proteins that enhance apoptosis are presented in Figure 3. Among these, only the expression of phosphoglycerate mutase 5 (PGAM5) was higher in untreated psoriatic keratinocytes than in healthy cells. As PGAM5 activates mainly caspase 9, it may be responsible for its activation in psoriasis. The UVB exposure decreased the levels of this protein and truncated BH3-interacting domain death agonist (tBid) expression, as well as BAX and PERK, but only in the case of psoriatic cells. Since BAX causes activation of caspase 9 and cytochrome c, the direction of changes in its expression corresponded to the changes in the expression of caspase 9 and cytochrome c while tBid is generated by proteolysis from Bid by caspase 8 and 9. Hence, proapoptotic UVB activity in the case of keratinocytes seems to be more related to the influence of irradiation on apoptosis inhibitors (Figure 3) than on positive regulators. On the other hand, CBD affected apoptosis by significant changes in its positive, as well as negative, regulator levels, particularly including decreased expression of p53 (Figure 3).

**Figure 4:**
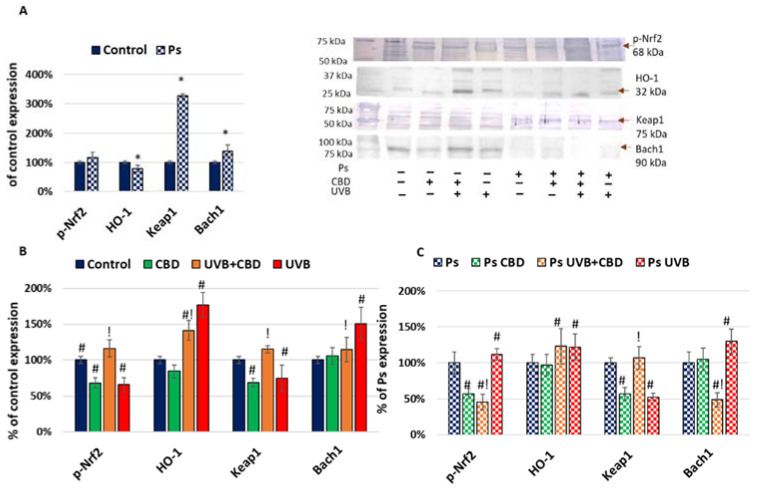
(**A**) Expression of proteins from Nrf2 pathway in keratinocyte cell lines from healthy subjects (Control; *n* = 5) and psoriatic patients (Ps; *n* = 5). Here, 100% corresponds to the level of protein in keratinocytes untreated with UVB or CBD. The mean values ± SD are presented with statistically significant differences: * between keratinocytes from patients with psoriasis vulgaris and healthy subjects, *p* < 0.05. (**B**) Expression of proteins from Nrf2 pathway in keratinocyte cell lines from healthy subjects (Control; *n* = 5) without treatment and after irradiation with UVB (60 mJ/cm^2^) and/or incubation with CBD (4 µM). Here, 100% corresponds to the level of protein in healthy keratinocytes untreated with UVB or CBD. The mean values ± SD are presented with statistically significant differences: # between keratinocytes treated with CBD, UVB and UVB+CBD and nontreated, *p* < 0.05; ! between keratinocytes treated with UVB+CBD and irradiated with UVB, *p* < 0.05. (**C**) Expression of proteins from Nrf2 pathway in keratinocyte cell lines from psoriatic patients (Ps; *n* = 5) without treatment and after irradiation with UVB (60 mJ/cm^2^) and/or incubation with CBD (4 µM). Here, 100% corresponds to the level of protein in psoriatic keratinocytes untreated with UVB or CBD. The mean values ± SD are presented with statistically significant differences: # between keratinocytes treated with CBD, UVB and UVB+CBD and nontreated, *p* < 0.05; ! between keratinocytes treated with UVB+CBD and irradiated with UVB, *p* < 0.05.

The Nrf2 is known to be one of the most important proteins involved in cell protection against oxidative stress, but it acts also as an inhibitor of apoptosis. In the case of psoriatic keratinocytes, the expression of the phosphorylated form of Nrf2 (p-Nrf2) was increased, although expression of its inhibitors was also enhanced, resulting in decreased HO-1 expression (Figure 4). The UVB decreased not only p-Nrf2 but also Keap1 expression, which resulted in higher expression of HO-1. This suggests activation of the Nrf2 pathway by UVB, which is in accordance with previous observations [26,27]. Similar changes were observed after CBD treatment. In cells treated by the combination of CBD and UVB, increased expression of p-Nrf2 was noticed if compared to CBD or UVB, while similar changes were also seen for Keap1, suggesting that the Nrf2 pathway was activated.

**Figure 5:**
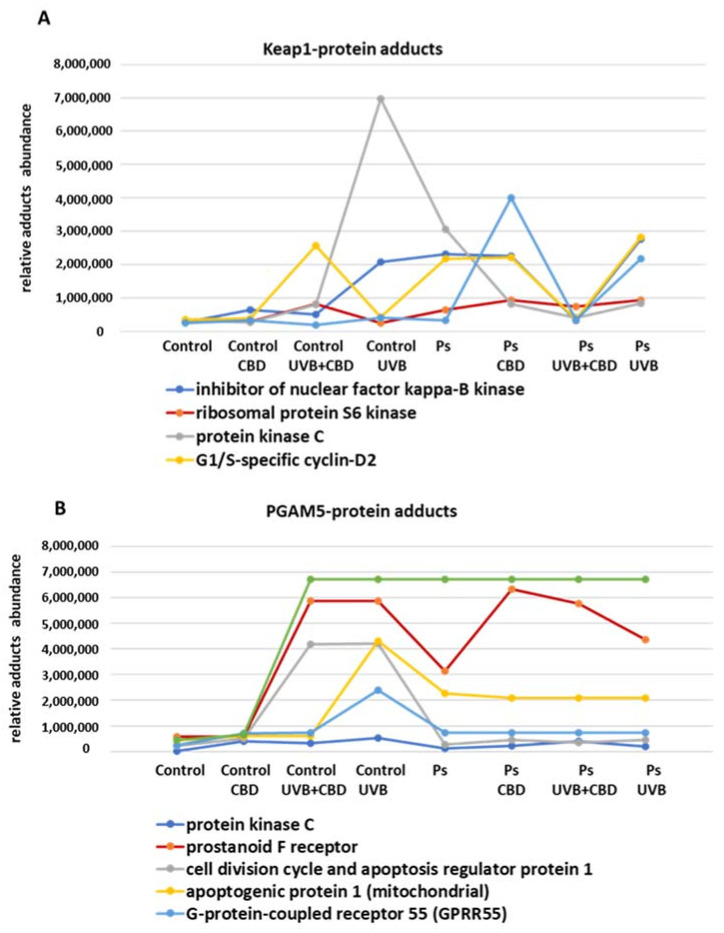
(**A**) Keap1–protein adducts in keratinocyte cell lines from healthy subjects (Control; *n* = 5) and psoriatic patients (Ps; *n* = 5) after irradiation with UVB (60 mJ/cm^2^) and incubation with CBD (4 µM). The point graph shows preliminary results of relative protein abundance of proteins making Keap-1 complexes in keratinocytes from healthy subjects and psoriatic patients (Ps) after UVB exposure and incubation with CBD. (**B**) PGAM5–protein adducts in keratinocyte cell lines from healthy subjects (Control; *n* = 5) and psoriatic patients (Ps; *n* = 5) after irradiation with UVB (60 mJ/cm^2^) and incubation with CBD (4 µM). The point graph shows preliminary results of relative protein abundance of proteins making PGAM5 complexes in keratinocytes from healthy subjects and psoriatic patients (Ps) after UVB exposure and incubation with CBD.

Regardless of the adducts with Nrf2, Keap1 may form adducts with other proteins that may be involved, inter alia, in the regulation of Nrf2 biological activity and apoptosis. The results of this study suggested that Keap1 forms complexes with IKKB, ribosomal protein S6 kinase, protein kinase C (PKC) and the protein p62 (Figure 5 and Figure S1).

It should be noticed that Keap1 had the highest expression in UVB- and CBD-treated psoriatic keratinocytes. Moreover, despite the fact that UVB and CBD caused similar changes in Keap1 expression, they nevertheless contributed to other metabolic modifications in cells irradiated with UVB vs. treated with CBD. Namely, the levels of Keap1–p62 adducts in the case of psoriatic cells and the levels of Keap1–PKC and Keap1–IKKB adducts in healthy cells have shown a differential response to UVB and CBD treatment. By interacting with IKKB, Keap1 can affect the extrinsic apoptotic pathway as an inhibitor of TNFα generation. In fact, the highest level of IKKB–Keap1 adducts is observed in UVB-irradiated healthy and psoriatic cells, and in these cells, the level of caspase 9, which is a marker of the intrinsic pathway, is reduced, confirming the inhibition of apoptosis. On the other hand, PKC, ribosomal protein S6 kinase and p62 are known to be inhibitors of the Nrf2 pathway. However, in the presented study, only the levels of the Keap1–p62 adducts were inversely correlated with the expression of Keap1, which suggests that they additionally increased the Keap1–Nrf2 interactions. Increased levels of Keap1–G1/S-specific cyclin D2 adducts were also observed in psoriasis, but their role in the regulation of apoptosis and the onset of psoriasis is not certain.

On the other hand, detected proteins considered as creating adducts with the mitochondrial serine/threonine-protein phosphatase (PGAM5) are those involved in the apoptosis regulation (such as p53, cell division cycle and apoptosis regulator protein 1 or apoptogenic protein 1), which act also within the cannabinoid system, like G protein-coupled receptor 55 (GPR55) and the prostanoid F receptor (Figure 5 and Figure S2). A higher level of protein adducts with the GPR55 receptor was observed in healthy cells after CBD administration, and UV exposure increased it even more, while UVB and CBD in combination abolished each other and eliminated the increased level of adducts. The prostanoid F receptor was unchanged by UVB but only in psoriatic keratinocytes, while its levels in healthy keratinocytes were increased by UVB, and its levels in both healthy and psoriatic cells were increased by the combined action of CBD and UVB. These results suggest that interactions between PGAM5 and other proteins play a role in the regulation of responses of the cells to cannabinoids, including CBD. In the case of proapoptotic proteins such as p53 and AP-1, it was observed that the level of their adducts in the psoriatic keratinocytes was generally higher than that in the group of healthy keratinocytes. Nevertheless, changes in the expression of these proteins in healthy cells were observed under the influence of UVB and CBD. In the case of AP-1, its level after UVB irradiation of keratinocytes was much higher than that after UVB+CBD treatment, which suggests that CBD has protective properties in this case.

**Figure 6:**
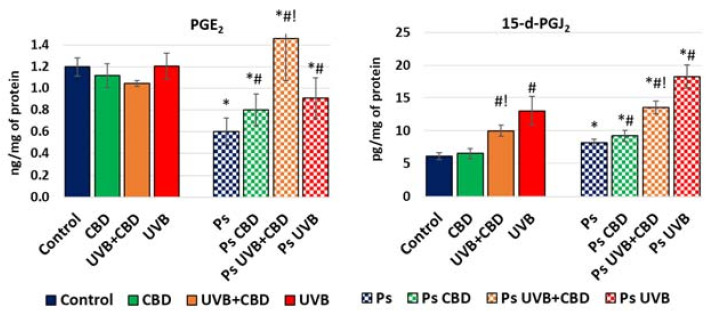
Levels of 15-d-PGJ_2_ and PGE_2_ in keratinocyte cell lines obtained from healthy subjects (Control; *n* = 5) and psoriatic patients (Ps; *n* = 5) without treatment and after irradiation with UVB (60 mJ/cm^2^) and/or incubation with CBD (4 μM). The mean values ± SD are presented with statistically significant differences: * between keratinocytes from patients with psoriasis vulgaris and healthy subjects, *p* < 0.05; # between treated keratinocytes (with CBD, UVB and UVB+CBD) and nontreated cells, *p* < 0.05; ! between keratinocytes treated with UVB+CBD and irradiated with UVB, *p* < 0.05.

Along with the analyzed proteins regulating apoptosis, this process can also be modulated by products of oxidative metabolism of membrane phospholipids generated under the influence of oxidative stress and inflammation. Among them, the most frequently mentioned as regulators of apoptosis are prostaglandins, including the proapoptotic 15-deoxy-prostaglandin J2 (15-d-PGJ2) and the antiapoptotic prostaglandin E2 (PGE2). The results of our study showed higher levels of 15-d-PGJ2 and lower levels of PGE2 in psoriatic ones than in healthy keratinocytes (Figure 6). UVB irradiation caused a significant increase in 15-d-PGJ2 levels in both healthy and psoriatic keratinocytes, and these levels were decreased after treatment with CBD. On the other hand, PGE2 levels increased after treatment with UVB or CBD and especially increased when both were used together, but only in psoriatic keratinocytes.

## 3. Discussion

Apoptosis is necessary to maintain skin homeostasis, so disturbances of the apoptotic process play an important role in many skin diseases, including psoriasis [20,28]. The pathophysiology of psoriasis is based on the increased proliferation of keratinocytes, which leads to the accumulation of these cells and thickening of the skin [29,30]. In order to prevent these changes, as well as the accumulation of infiltrating leukocytes within the psoriatic lesions, UVB phototherapy is used [31].

### 3.1. Apoptosis in Psoriatic Keratinocytes

Apoptosis is initiated by various proapoptotic pathways that are under the control of the AKT/p38 system [32], which initiates the activation of initiating caspases [5], notably caspases 2, 8, 9 and 10, which then activate the executive caspases (caspases 3, 6 and 7) [33]. In healthy cells and depending on the exogenous factors used, the increased expression of antiapoptotic p-AKT automatically lowers the proapoptotic p-p38 level and vice versa. However, this system is completely disturbed in psoriatic keratinocytes, where UVB or CBD treatments will increase p-p38 expression independent of p-AKT expression. Moreover, the results of this study indicate that psoriasis development following UVB irradiation is associated with activation of caspases 8 and 9, which then activate the executive caspase 3. It is known that caspase 9 is also activated by the protein Bak and BAX, which under physiological conditions remain in complex with antiapoptotic proteins [34]. However, various proapoptotic proteins, such as p53, break down these complexes and activate Bak and BAX [6,7,35]. In the case of psoriatic keratinocytes, induction of the extrinsic apoptotic pathway promotes activation of caspase 8, which may further activate caspases 3 and 7 [36,37]. It should be mentioned that activation of caspase 8 begins with the interaction of TNFα with its membrane receptors, and previous studies have demonstrated increased levels of this inflammatory cytokine in psoriatic keratinocytes [38]. It is known that the prostaglandin 15-d-PGJ_2_ induces the extrinsic pathway by stimulation of TNFα biosynthesis, which can be prevented by TNFR1 antagonists [39]. The proapoptotic effect of this prostaglandin was observed in various cells, including human keratinocytes obtained from healthy individuals [39,40,41]. Increased levels of prostaglandins usually accompany inflammation, as has also been observed in psoriatic leukocytes [42,43]. Since a higher level of proapoptotic 15-d-PGJ_2_ was observed in psoriatic keratinocytes when the antiapoptotic PGE_2_ level was lowered, the imbalance between these prostaglandins seems to play an important role in the regulation of apoptosis in psoriatic cells.

However, the observed increase in PGAM5 level suggests this may be the reason for the activation of the intrinsic proapoptotic pathway, as overexpression of this protein is associated with caspase 9 activation [44]. Moreover, the suggested increase in PGAM5–p53 adduct level in psoriatic keratinocytes may affect caspase 2 because p53 is an important activator of proapoptotic proteins [45]. However, the p53 and PGAM5 interactions have so far not been sufficiently studied, so it can be assumed that p53 can also activate PGAM5 as well as caspases 3, 8 and/or 9. On the other hand, lower PI3K expression indicates that the antiapoptotic axis of PI3K–AKT was inhibited in psoriatic keratinocytes, which may explain why psoriatic keratinocytes are more prone to apoptosis than keratinocytes from healthy subjects. The results of the current study and previous studies [46] indicate that the antioxidant capacity in psoriatic keratinocytes is reduced, which may also affect the apoptosis process. The prominent increase in the inhibitors of Nrf2, such as Keap1 and Bach1, observed in our study may lead to a decrease in the expression of HO-1, considered as a biomarker of transcriptional effectiveness of Nrf2. Furthermore, protein kinase C (PKC), p62 and the ribosomal protein S6 kinase are involved in the interactions between Nrf2 and Keap1, and we observed increased levels of adducts of these proteins with Keap1 in psoriatic cells. This may, in part, explain why the expression of HO-1 is lower in psoriatic keratinocytes despite similar Nrf2 expression. Besides, PKC is believed to activate Nrf2 by phosphorylation of its Ser40 residue leading to the dissociation of Keap1 from Nrf2 [47]. This would agree with the results of our study indicating that PKC forms a complex with Keap1 consequently enhancing the transcriptional activity of Nrf2.

### 3.2. Effects of CBD or UVB Radiation on Apoptosis of Keratinocytes

It is known that UVB irradiation used in psoriasis phototherapy increases the level of proinflammatory cytokines and biomarkers of oxidative stress in keratinocytes [31,48,49]. The effectiveness of UVB phototherapy is mostly due to the induction of apoptosis in leukocytes infiltrating the skin [50,51]. Still, UVB phototherapy affects not only leukocytes but also keratinocytes, intensifying apoptosis of both healthy and diseased cells The influence of CBD on apoptosis is less clear [52,53], as CBD may act as both a pro- and antiapoptotic factor, depending on the type of cells, their receptors and their metabolic profile [5]. Moreover, CBD has been found as a reducing factor of mitochondrial membrane potential which in cancer cells results in lower ATP production and apoptosis stimulation [54].

The UV irradiation enhances ROS production and activates proinflammatory pathways, including biosynthesis of prostaglandins and “the death ligands”, especially TNFα, which activates the extrinsic pathway of apoptosis through caspase 8 [55]. Other authors also point to the role of caspases 3 and 8 in the apoptosis of keratinocytes from healthy people after exposure to UVB irradiation [56,57], but so far there have been no data on the response of psoriatic keratinocytes. The results of our study indicate that UVB leads mainly to the activation of the extrinsic apoptotic pathway in healthy and psoriatic cells, likely due to its impact on the level of proapoptotic 15-d-PGJ_2_. CBD had similar effects in psoriatic cells, although less pronounced, being ineffective in healthy cells, indicating its possible use for more selective treatment of psoriasis.

**Figure 7:**
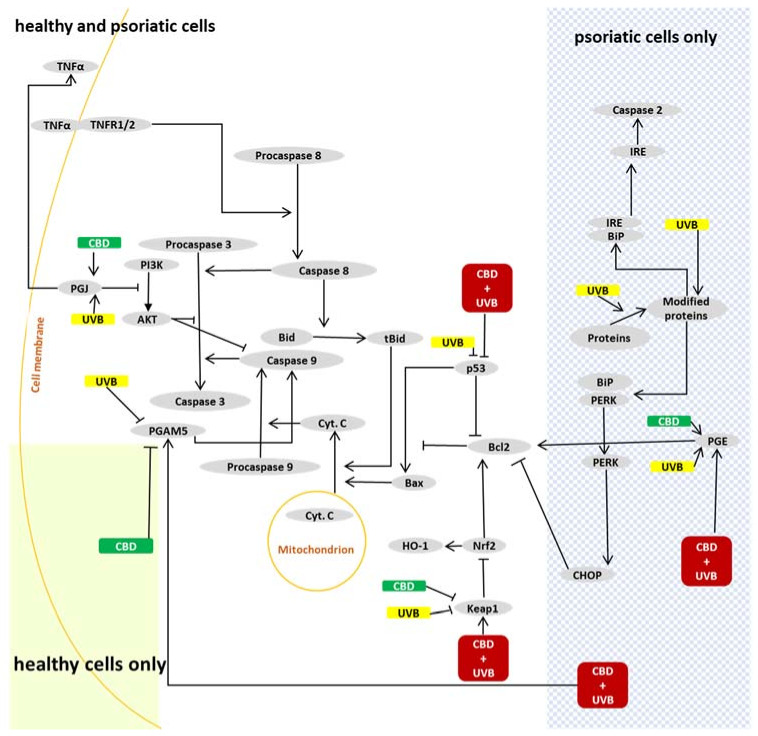
The most important pathways involved in modulation of apoptosis by CBD, UVB or CBD+UVB in psoriatic and healthy keratinocytes.

Independent of the effects on the extrinsic apoptotic pathway, UVB may modify ER stress-initiated apoptosis. The initiator of this pathway is mainly caspase 2, but it can also be initiated by ROS or lipid peroxidation products and thus modified proteins [58] (Figure 7). However, our study did not indicate caspase 2 activation through the PERK–CHOP axis, since the PERK and CHOP levels were not increased by UVB irradiation. Previous studies on keratinocytes exposed to UV radiation also showed no changes in CHOP expression [59]. Thus, it may suggest that caspase 2 was maybe activated by the IRE protein, especially as IRE is known to be activated by UVB in keratinocytes [58,60].

The results of this study have also shown that UVB irradiation reduced the expression of p53 in healthy keratinocytes. According to our preliminary proteomic studies, this may be related to the increased level of PGAM5–p53 adducts. In the case of CBD, its influence on p53 was similar but was likely caused by a different mechanism. It is known that CBD can induce the synthesis of MDM2, a negative regulator of p53 in keratinocytes [61]. Moreover, since p53 is an activator of the intrinsic apoptotic pathway, changes in its expression may be responsible for decreased caspase 9, which was observed in psoriatic cells after CBD or UVB irradiation, suggesting the inhibition of the intrinsic apoptotic pathway.

Similarities observed for both healthy and psoriatic cells may be caused by the lack of changes of caspase 3 and caspase 9 levels, due to compensatory protective mechanisms based on the PI3K–AKT and Nrf2 pathways or because of change in the Bcl2/BAX ratio. According to our findings, the PI3K–AKT axis was not activated following UVB or CBD treatment. However Bcl2 expression was increased in psoriatic keratinocytes, and it is likely that Bcl2 protected these cells despite activated proapoptotic pathways. Expression of Bcl2 also depends on the biological activity of Nrf2 [62], but there were different changes in Bcl2, Nrf2 and HO-1 expression after CBD treatment, which suggests that Bcl2 expression is regulated not only by Nrf2 but also by other factors. Higher levels of PGE_2_ may also be a reason for the increased activity of COX2, which has been observed previously in different cells after UVB radiation [42,63,64]. In our study, UVB increased PGE_2_ levels but only in psoriatic keratinocytes, corresponding to changes in Bcl2 expression.

It is known that UVB irradiation may change the expression of cannabinoid receptors, which in turn leads to changes in the levels of ROS and TNFα [65]. We have previously described higher expression of pro-oxidative and proinflammatory cannabinoid receptor 1 (CB1) in psoriatic keratinocytes, which shows that UV increases inflammation and apoptosis [19]. Furthermore, since *CB1/2*^−/−^ animals are resistant to UV irradiation-induced inflammation, it seems that endocannabinoid signaling plays an important role in UV-induced signaling pathways [66]. This may suggest that keratinocytes developed a genuine cannabinoid-receptor pattern, making them sensitive to the antioxidant effects of cannabinoids. That is in agreement with previous findings showing that CBD increases cannabinoid signaling in healthy and psoriatic keratinocytes [19].

### 3.3. Effects of CBD on Apoptosis in UVB-Irradiated Keratinocytes

Metabolic changes associated with the UVB phototherapy of psoriatic keratinocytes suggest options for adjuvant treatments that would support more selective effects of phototherapy and perhaps even protect healthy keratinocytes. Our previous findings have shown that CBD shows proapoptotic effects for psoriatic keratinocytes, similar to UVB radiation applied alone. For cells simultaneously treated with UVB and CBD, UVB irradiation increased the expression of CB1 and CB2 receptors, while CBD additionally increased the expression of CB2 in healthy cells, but not in psoriatic keratinocytes [19]. This difference in cannabinoid receptor response may explain why CBD was protective against UV-induced apoptosis in healthy keratinocytes in our study. It is known that CB2 receptors are responsible for lowering ROS and TNFα production, and therefore an increase in their expression would have antioxidant and anti-inflammatory effects, which may be responsible for differential responses of psoriatic and healthy keratinocytes to CBD treatment. If so, AKT seems to be of particular importance, since activation of the CB2 receptor leads to the activation of this kinase [67], which is an important inhibitor of apoptosis. Furthermore, p38 can be activated by CB1 agonists, while it is inhibited by CB2 agonists [68,69], showing that the effects of cannabinoids on the expression of p38 depend on the receptor profile of the cells. Conversely, a decrease in 15-d-PGJ_2_ level after CBD treatment could lead to a decrease in p-p38 expression, as was observed in our study. Notably, CBD reduced 15-d-PGJ_2_ level and p-p38 expression to much lower levels in healthy cells compared to psoriatic keratinocytes. While CBD had no impact on PGE_2_ expression in healthy cells, it increased the level of this prostaglandin in psoriatic keratinocytes. Hence, PGE_2_ expression was highest in psoriatic keratinocytes exposed to the combined treatment with UVB and CBD. On the other hand, levels of 15-d-PGJ_2_ were lower in these cells compared with those receiving only UVB treatment. This can be caused by CBD-dependent decreases in COX-2 activity, which therefore decreases the synthesis of prostaglandin, as observed in the case of PGJ_2_ [70]. On the other hand, UV increases the expression of PGE synthase [71], which therefore favors the synthesis of PGE_2_ when CBD prevents the synthesis of PGJ_2_. Moreover, CBD in general increases PGE_2_, but the mechanism of action is not known. Therefore, an imbalance between 15-d-PGJ_2_ and PGE_2_ prostaglandins, as well as a lack of AKT activation, with a higher expression of p-p38 could eventually lead to higher expression of caspases in psoriatic keratinocytes than in healthy cells treated with CBD. This would confirm the activation of various apoptotic pathways in psoriatic keratinocytes and offer a possible explanation for the differential antiapoptotic effects of CBD for healthy and psoriatic keratinocytes observed after UVB irradiation in vitro.

The results of this study indicate that CBD significantly influences the extrinsic apoptotic pathway because the expression of caspase 8 was significantly reduced by CBD. Interestingly, this is opposite to the increased expression of caspase 8 after UVB irradiation of psoriatic keratinocytes. This may also be effective for the regulation of apoptosis in psoriatic plaques, which should be tested in the future.

The differential response of psoriatic and healthy keratinocytes might also occur in vivo due to the anti-inflammatory activities of CBD that may reduce the TNFα biosynthesis, which can inhibit the extrinsic apoptotic pathway. Moreover, CBD ameliorates the direct influence of TNFα on keratinocytes [72,73]. In addition, CBD may reduce the expression of the Nrf2 inhibitor Keap1 by inducing the formation of Keap1–p62 adducts that have protective effects. Furthermore, since p62 is responsible for the activation of Nrf2 [74,75], the CBD-induced formation of Keap1–p62 adducts could lead to the dissociation of Keap1 from the Nrf2–Keap1 complex and the consequential biosynthesis of antioxidants due to Nrf2 transcription activity. Therefore, p62 may be responsible for the antioxidant properties of CBD in UV-irradiated cells. Because CBD itself has antioxidant effects, its use reduces redox imbalance and inflammation. This could result in a relatively selective reduction of apoptosis in healthy keratinocytes, as was observed in the study.

Another explanation for the differential effects of CBD for healthy and psoriatic keratinocytes is that interactions between PGAM5 and GPR55, as well as between PGAM5 and the prostanoid F receptor, were observed after UV irradiation. However, we did not see the adducts of PGAM5 and RIPK3 (receptor-interacting serine/threonine-protein kinase 3), which is a member of the TNFR1 signaling complex, so PGAM5 was probably not involved in TNFα-induced cell death. Because CBD is an agonist of GPR55, the CBD metabolites are prostanoid F receptor agonists that may be important for the regulation of PGAM5. This assumption seems credible because PGAM-5 can be inactivated by the abovementioned receptors, which can inhibit apoptosis [76].

The observed protection of healthy keratinocytes by CBD against UV-induced apoptosis could be beneficial for the therapy of psoriasis. However, the results of this study indicate that CBD may partially counteract the effects of phototherapy. On the other hand, as CBD may influence the differentiation of keratinocytes by downregulating genes involved in the keratinization process, CBD may act against the creation of psoriatic plaques through this mechanism [77]. Moreover, previous research has demonstrated that the beneficial effects of phototherapy appear to be due to its effect on reducing the apoptosis of skin-infiltrating Th1 lymphocytes more than because of its direct effects on keratinocytes [51]. While CBD is a proapoptotic factor for lymphocytes [78,79], CBD could even increase such indirect effects of UV phototherapy.

## 4. Materials and Methods

### 4.1. Material

#### 4.1.1. Patient/Donor Selection and Cell Line Acquisition

Skin tissues were collected from five untreated patients with a diagnosis of psoriasis vulgaris (2 men and 3 women; age range 28–54 years, mean 40) and five healthy individuals (sex- and age-matched individuals forming a control group; age range 25–51 years, mean 39). Eligible patients were those diagnosed with plaque psoriasis for at least 6 months with lesions affecting at least 10% of total body surface area. The severity of psoriasis was assessed using the Psoriasis Area and Severity Index (PASI) score (median 17; range 13–21). None of the patients or healthy subjects received topical, injectable or oral medications during the 4 weeks before the study. Individuals whose history indicated any other disorders were excluded from the study. None of the participants were smokers. The study was conducted in accordance with the Declaration of Helsinki, and the protocol for the collection of skin samples was approved by the Local Bioethics Committee in Medical University of Bialystok (Poland), No. R-I-002/289/2017. Written informed consent was obtained from all participants.

Skin biopsies were obtained from active psoriasis lesions from the elbow or knee area. The samples were collected using the classical surgical method. In healthy controls, the nevus was collected together with the surrounding tissue. After skin decontamination, infiltration anesthesia with 1% lignocaine was applied, and then skin fragments of approximately 5–8 mm were collected. Samples immediately after biopsy were destined for histopathological examination (hematoxylin–eosin staining); data were shown previously [80]. The rest of the sample was washed in phosphate-buffered saline (PBS) supplemented with 50 U/mL penicillin and 50 μg/mL streptomycin and then was incubated overnight at 4 °C in dispase solution 1 mg/mL (Gibco, Grand Island, NY, USA) to separate epidermis from dermis. All abovementioned cell culture reagents as well as consumables were obtained from Gibco (Gibco, Grand Island, NY, USA). Following incubation, the epidermis was digested by a 20 min incubation at 37 °C using trypsin/ethylenediamine tetraacetic acid (EDTA). Separated keratinocytes were washed and resuspended in growing medium from Keratinocyte Growth Kit (Gibco, Grand Island, NY, USA), which contained Keratinocyte Serum-Free Medium supplemented with 10% fetal bovine serum (10%), 5 g/L epidermal growth factor EGF 1–53 (5 g/L), penicillin (50 U/mL) and streptomycin (50 g/mL). Amplified keratinocytes from each donor were separate cell lines treated during the experiment.

#### 4.1.2. Cell Culture and Treatment

Keratinocyte cell lines (healthy and psoriatic) were cultured in a growing medium described above. The cells were cultured under standard conditions (humidified atmosphere of 5% CO_2_ at 37 °C) until the 4th passage. Cell culture purity and uniformity were controlled based on the keratinocyte morphology observation. After reaching 80% confluency, keratinocytes were washed with PBS (37 °C). The cells were irradiated with UVB on ice, to avoid heat stress and oxidation of medium components, at a distance of 15 cm from the assembly of 6 lamps (Bio-Link Crosslinker BLX 365/312; Vilber Lourmat, Germany) at 6 W each, corresponding to 4.08 mW/cm^2^ (312 nm and the total radiation dose 60 mJ/cm^2^). The wavelength used corresponds to the wavelength of the UVB radiation used in narrow-band phototherapy of psoriasis [81]. The 70% cell viability measured by the MTT assay [82] was an indicator of the dose selection. To analyze the effect of CBD on these cells, a suspension of CBD in ethanol was added to a final concentration of 4 µM (the final concentration of ethanol was 0.3%). The concentration of CBD used did not change the morphology and proliferation of keratinocytes [75,83], nor did it affect cell viability measured with the MTT assay [82] (data not shown).

The studies were carried out in the following groups:

Healthy keratinocytes, divided into further subgroups:

- Control keratinocytes (Control)—cultured in the medium described above;
- Control keratinocytes treated with CBD (CBD)—cells cultured for 24 h in the medium described above supplemented with 4 µM CBD;
- Control keratinocytes irradiated with UVB (UVB)—cells treated with UVB (60 mJ/cm^2^) and next cultured for 24 h in the medium described above;
- Control keratinocytes irradiated with UVB and treated with CBD (UVB+CBD)—cells treated with UVB and next cultured for 24 h in the medium described above supplemented with 4 µM CBD as in CBD group.

Psoriatic keratinocytes:

■ Psoriatic keratinocytes (Ps)—cultured in the medium described above;
■ Psoriatic keratinocytes treated with CBD (Ps CBD)—cells cultured for 24 h in the medium described above supplemented with 4 µM CBD;
■ Psoriatic keratinocytes irradiated with UVB (Ps UVB)—cells treated with UVB and next cultured for 24 h in the medium described above;
■ Psoriatic keratinocytes irradiated with UVB and treated with CBD (Ps UVB+CBD)—cells treated with UVB and next cultured for 24 h in the medium described above supplemented with 4 µM CBD as in Ps CBD group.

After incubation, cells were frozen at −86 °C for examination of protein expression, identification of protein adducts and determination of prostaglandin levels.

### 4.2. Methods

#### 4.2.1. Determination of Protein Expression

Western blot analysis of keratinocyte protein expression was performed according to the protocol described in Eissa and Seada [84]. Keratinocytes were sonicated and centrifuged (15,000× *g*, 30 min, 4 °C) to obtain a protein fraction that was used for the detection of specific proteins by Western blot. Normalized amount of proteins (measured using Bradford assay [85]) was electrophoretically separated on 10% polyacrylamide gels, transferred to nitrocellulose membranes (0.2 µm pore size), blocked with 5% milk for 1 h (Bio-Rad Laboratories Inc., Hercules, CA, USA) and then washed four times with TBS-T buffer. Washed membranes were incubated overnight with the following primary antibodies: rabbit against caspase 2 (Abcam, Cambridge, MA, USA), caspase 9 (Assay Biotechnology, Fremont, CA, USA) and caspase 8 (Novus Biologicals, Centennial, CO, USA); PI3K (Cell Signaling Technology, Inc., Danvers, MA, USA); PGAM5, HO-1, Bach1 and Keap1 (Sigma-Aldrich, St. Louis, MO, USA); p-PERK (phospho-Thr980) (R&D Systems, Inc., Minneapolis, MN, USA); p-p38 (phospho-Tyr182); p-AKT (phospho-Ser473); p-Nrf2 (phospho-Ser40); tBid (Bioss Antibodies Inc., Woburn, MA, USA); mouse against Bcl2, Ref1, p53 and cytochrome c (Santa Cruz Biotechnology, Dallas, TX, USA); BAX and CHOP (Invitrogen, Thermo Fisher Scientific, Inc., Cleveland, OH, USA); β-actin (Sigma-Aldrich, St. Louis, MO, USA); and goat against caspase 3 (R&D Systems, Inc., Minneapolis, MN, USA). After incubation, the membranes were then washed four times with TBS-T and incubated for 2 h with corresponding polyclonal alkaline phosphatase secondary antibodies (Sigma-Aldrich, St. Louis, MO, USA). Protein bands were visualized using the BCIP/NBT liquid substrate system (Sigma-Aldrich, St. Louis, MO, USA). To compare the protein concentrations between samples, each band intensity was estimated using VersaDoc System and Quantity One software (Bio-Rad Laboratories Inc., Hercules, CA, USA). The results were expressed as a percentage of the expression determined in the control cells.

#### 4.2.2. Isolation of Keratinocyte Protein Adducts

To estimate the level of proteins creating adducts with Keap1 or PGAM5, these proteins were immunoprecipitated with all molecules that formed strong enough bonds with them to be considered as adducts. For this reason, cell lysates were precleaned with protein A agarose to remove molecules that could nonspecifically react with this substance. Protein A agarose was removed by 1 min centrifugation (10,000× *g*, 4 °C). Next, the primary antibody against Keap1 (1:1000; Santa Cruz Biotechnology, Santa Cruz, CA, USA) was added, and samples were incubated for 1 h in 4 °C. To precipitate the proteins bound with antibodies, protein A agarose was added and incubated overnight. The next day, samples were centrifuged (10 min, 10,000× *g*, 4 °C), and the obtained pellet was received as Keap1 and proteins immunoprecipitated with Keap1. The remaining supernatant was incubated with primary antibody against PGAM5 (1:1000; Sigma-Aldrich, St. Louis, MO, USA), and the proteins that bind to PGAM5 were precipitated in an identical manner as previously.

#### 4.2.3. Protein Adduct Identification by MS/MS

Obtained mixtures of proteins were mixed 1:1 with a sample loading buffer (Laemmli buffer containing 5% 2-mercaptoethanol), heated at 100 °C for 7 min and separated using 10% Tris-Glycine SDS-PAGE gels. After electrophoretic separation, the gels were stained overnight with Coomassie Brilliant Blue R-250. All bands were cut from the gels, and contained proteins were reduced with 10 mM DTT, alkylated by incubation with 50 mM iodoacetamide and in-gel digested overnight with trypsin (Promega, Madison, WI, USA). The isolated peptide mixture was extracted from the gel, dried and dissolved in 5% acetonitrile with 0.1% formic acid. The final peptide mixture was separated using an Ultimate 3000 high-performance liquid chromatography (HPLC) system (Dionex, Idstein, Germany) on a 150 mm × 0.075 mm PepMap RSLC capillary analytical C18 column with 2 µm particle size (Dionex, LC Packings). Eluted peptides were analyzed using a Q Exactive HF mass spectrometer with an electrospray ionization source (ESI) (Thermo Fisher Scientific, Bremen, Germany). The conditions of the analysis by LC-MS/MS for peptide identification have been described in detail previously. Obtained raw data were analyzed using Proteome Discoverer 2.0 (Thermo Fisher Scientific) and searched against the UniProtKB-SwissProt database (taxonomy: Homo sapiens, release 2020-04). For the identification of proteins, the following search parameters were used: peptide mass tolerance set to 10 ppm, MS/MS mass tolerance set to 0.02 Da, up to two missed cleavages allowed, cysteine carboxymethylation and methionine oxidation set as dynamic modifications. Protein label-free quantification was performed according to the signal intensities of the precursor ions. Results from individual protein quantification were normalized by the sample sum, log-transformed and scaled to the row mean following Z-scoring. Data were visualized as Heatmaps created with free available MetaboAnalyst 4.0 software (www.metaboanalyst.ca, accessed on 24 May 2021). As there is a lack of validation at the level of both adduct isolation and analysis of the selected protein levels by MS/MS, results of these examinations are considered as preliminary, and only general assumptions were drawn according to them.

#### 4.2.4. Determination of Prostaglandin Levels

The lipid fraction of sonicated keratinocytes was isolated using the SPE method (Waters Oasis HLB 3cc). Eicosanoids, from the above fraction, were separated on Zorbax Eclipse Plus C18 analytical column (2.1 × 100 mm, 1.8 µm particle size). Prostaglandin E_2_ (PGE_2_) and 15-deoxy-delta-12,14-prostaglandin J_2_ (15-d-PGJ_2_) were determined using LC-MS/MS (LC-MS 8060, Shimadzu, Kyoto, Japan). Electrospray ionization (ESI) in negative mode was used for multiple reaction monitoring (MRM) and quantification of analytes. PGD_2_-d_4_, PGF2α-d4 and PGD2-d4 were used as internal standards for quantification. The precursors to the product ion transition were as follows: m/z 351.3→271.20 for PGE_2_ and m/z 315.2→271.2 for 15-d-PGJ_2_. The amounts of prostaglandins were expressed in pg or ng/mg protein (protein levels were examined using Bradford assay [85]).

### 4.3. Statistical Analysis

Statistical analysis between groups was performed using one-way ANOVA test using Statistica software (Statistica 13.3 StatSoft Polska Sp. z o.o., Kraków, Poland), and statistically significant differences, for *p* < 0.05, are shown in the figures.

## 5. Conclusions

Overall, our study showed that the development of psoriasis is accompanied by increased apoptosis through the internal and external pathways, both of which are further exacerbated by CBD or UVB irradiation. The results of this study suggest that these changes may be the result of PGE_2_-induced modification of the Bcl2/BAX ratio. However, irradiation of psoriatic keratinocytes with UVB also activates the ER stress-induced pathway. This is not seen after treatment with CBD. Moreover, the differences in expression of p53, p38 and caspase 8 observed between healthy and psoriatic keratinocytes after combined CBD and UVB treatment indicate that CBD protects healthy keratinocytes more than psoriatic cells. Given its anti-inflammatory and antioxidant effects, CBD may be considered a potentially adjuvant option in the integrated biomedical treatment of psoriasis. Considering the conditions and results of this study, the topical application of CBD ointment on skin lesions for 24 h after exposure to UVB rays seems to be particularly beneficial.

## Data Availability

The data presented in this study are available upon request.

## Supplementary Materials

The following are available online at https://www.mdpi.com/article/10.3390/ijms22189956/s1.

## References

[1] Furue K., Ito T., Tsuji G., Nakahara T., Furue M. (2020). The CCL20 and CCR6 Axis in Psoriasis. Scand. J. Immunol..

[2] D’Arcy M.S. (2019). Cell Death: A Review of the Major Forms of Apoptosis, Necrosis and Autophagy. Cell Biol. Int..

[3] Pistritto G., Trisciuoglio D., Ceci C., Garufi A., D’Orazi G. (2016). Apoptosis as Anticancer Mechanism: Function and Dysfunction of Its Modulators and Targeted Therapeutic Strategies. Aging (Albany NY).

[4] Sano R., Reed J.C. (2013). ER Stress-Induced Cell Death Mechanisms. Biochim. Biophys. Acta (BBA)—Mol. Cell Res..

[5] Wójcik P., Žarković N., Gęgotek A., Skrzydlewska E. (2020). Involvement of Metabolic Lipid Mediators in the Regulation of Apoptosis. Biomolecules.

[6] Wu C.-C., Bratton S.B. (2013). Regulation of the Intrinsic Apoptosis Pathway by Reactive Oxygen Species. Antioxid. Redox Signal..

[7] Viant C., Guia S., Hennessy R.J., Rautela J., Pham K., Bernat C., Goh W., Jiao Y., Delconte R., Roger M. (2017). Cell Cycle Progression Dictates the Requirement for BCL2 in Natural Killer Cell Survival. J. Exp. Med..

[8] Bohnert K.R., McMillan J.D., Kumar A. (2018). Emerging Roles of ER Stress and Unfolded Protein Response Pathways in Skeletal Muscle Health and Disease. J. Cell Physiol..

[9] Bendavit G., Aboulkassim T., Hilmi K., Shah S., Batist G. (2016). Nrf2 Transcription Factor Can Directly Regulate MTOR. J. Biol. Chem..

[10] Luo J.-L., Kamata H., Karin M. (2005). IKK/NF-ΚB Signaling: Balancing Life and Death—A New Approach to Cancer Therapy. J. Clin. Investig..

[11] Lee D.-F., Kuo H.-P., Liu M., Chou C.-K., Xia W., Du Y., Shen J., Chen C.-T., Huo L., Hsu M.-C. (2009). KEAP1 E3 Ligase-Mediated Down-Regulation of NF-ΚB Signaling by Targeting IKKβ. Mol. Cell.

[12] Kim J.-E., You D.-J., Lee C., Ahn C., Seong J.Y., Hwang J.-I. (2010). Suppression of NF-KappaB Signaling by KEAP1 Regulation of IKKbeta Activity through Autophagic Degradation and Inhibition of Phosphorylation. Cell Signal..

[13] Tami Wong B.S., Leon Hsu B.A., Wilson Liao M.D. (2013). Phototherapy in Psoriasis: A Review of Mechanisms of Action. J. Cutan. Med. Surg..

[14] Lee C.-H., Wu S.-B., Hong C.-H., Yu H.-S., Wei Y.-H. (2013). Molecular Mechanisms of UV-Induced Apoptosis and Its Effects on Skin Residential Cells: The Implication in UV-Based Phototherapy. Int. J. Mol. Sci..

[15] Chiurchiù V., Cencioni M.T., Bisicchia E., Bardi M.D., Gasperini C., Borsellino G., Centonze D., Battistini L., Maccarrone M. (2013). Distinct Modulation of Human Myeloid and Plasmacytoid Dendritic Cells by Anandamide in Multiple Sclerosis. Ann. Neurol..

[16] Souza M.C., Rosas E.C. (2018). Cannabinoid Receptors as Regulators of Neutrophil Activity in Inflammatory Diseases. Neutrophils.

[17] Martinelli G., Magnavacca A., Fumagalli M., Dell’Agli M., Piazza S., Sangiovanni E. (2021). Cannabis Sativa and Skin Health: Dissecting the Role of Phytocannabinoids. Planta Med..

[18] Baswan S.M., Klosner A.E., Glynn K., Rajgopal A., Malik K., Yim S., Stern N. (2020). Therapeutic Potential of Cannabidiol (CBD) for Skin Health and Disorders. Clin. Cosmet. Investig. Dermatol..

[19] Jarocka-Karpowicz I., Biernacki M., Wroński A., Gęgotek A., Skrzydlewska E. (2020). Cannabidiol Effects on Phospholipid Metabolism in Keratinocytes from Patients with Psoriasis Vulgaris. Biomolecules.

[20] Elmore S. (2007). Apoptosis: A Review of Programmed Cell Death. Toxicol. Pathol..

[21] Dondelinger Y., Aguileta M.A., Goossens V., Dubuisson C., Grootjans S., Dejardin E., Vandenabeele P., Bertrand M.J.M. (2013). RIPK3 Contributes to TNFR1-Mediated RIPK1 Kinase-Dependent Apoptosis in Conditions of CIAP1/2 Depletion or TAK1 Kinase Inhibition. Cell Death Differ..

[22] Atalay S., Gęgotek A., Wroński A., Domigues P., Skrzydlewska E. (2021). Therapeutic Application of Cannabidiol on UVA and UVB Irradiated Rat Skin. A Proteomic Study. J. Pharm. Biomed. Anal..

[23] Atalay S., Dobrzyńska I., Gęgotek A., Skrzydlewska E. (2020). Cannabidiol Protects Keratinocyte Cell Membranes Following Exposure to UVB and Hydrogen Peroxide. Redox Biol..

[24] Siddiqui W.A., Ahad A., Ahsan H. (2015). The Mystery of BCL2 Family: Bcl-2 Proteins and Apoptosis: An Update. Arch. Toxicol..

[25] Shrivastava A., Kuzontkoski P.M., Groopman J.E., Prasad A. (2011). Cannabidiol Induces Programmed Cell Death in Breast Cancer Cells by Coordinating the Cross-Talk between Apoptosis and Autophagy. Mol. Cancer Ther..

[26] Knatko E.V., Ibbotson S.H., Zhang Y., Higgins M., Fahey J.W., Talalay P., Dawe R.S., Ferguson J., Huang J.T.-J., Clarke R. (2015). Nrf2 Activation Protects against Solar-Simulated Ultraviolet Radiation in Mice and Humans. Cancer Prev. Res. (Phila).

[27] Hirota A., Kawachi Y., Itoh K., Nakamura Y., Xu X., Banno T., Takahashi T., Yamamoto M., Otsuka F. (2005). Ultraviolet A Irradiation Induces NF-E2-Related Factor 2 Activation in Dermal Fibroblasts: Protective Role in UVA-Induced Apoptosis. J. Investig. Dermatol..

[28] Jia H.-Y., Zhang K., Lu W.-J., Xu G.-W., Zhang J.-F., Tang Z.-L. (2019). LncRNA MEG3 Influences the Proliferation and Apoptosis of Psoriasis Epidermal Cells by Targeting MiR-21/Caspase-8. BMC Mol. Cell Biol..

[29] Huang T.-H., Lin C.-F., Alalaiwe A., Yang S.-C., Fang J.-Y. (2019). Apoptotic or Antiproliferative Activity of Natural Products against Keratinocytes for the Treatment of Psoriasis. Int. J. Mol. Sci..

[30] Kim J., Nadella P., Kim D.J., Brodmerkel C., Correa da Rosa J., Krueger J.G., Suárez-Fariñas M. (2015). Histological Stratification of Thick and Thin Plaque Psoriasis Explores Molecular Phenotypes with Clinical Implications. PLoS ONE.

[31] Zhang P., Wu M.X. (2018). A Clinical Review of Phototherapy for Psoriasis. Lasers Med. Sci..

[32] Rane M.J., Song Y., Jin S., Barati M.T., Wu R., Kausar H., Tan Y., Wang Y., Zhou G., Klein J.B. (2010). Interplay between Akt and P38 MAPK Pathways in the Regulation of Renal Tubular Cell Apoptosis Associated with Diabetic Nephropathy. Am. J. Physiol. Ren. Physiol..

[33] Li J., Yuan J. (2008). Caspases in Apoptosis and Beyond. Oncogene.

[34] Rupniewska Z., Bojarska-Junak A. (2004). Apoptosis: Mitochondrial membrane permeabilization and the role played by Bcl-2 family proteins. Postepy Hig. Med. Dosw. (Online).

[35] Li X., Miao X., Wang H., Xu Z., Li B. (2015). The Tissue Dependent Interactions between P53 and Bcl-2 in Vivo. Oncotarget.

[36] Lamkanfi M., Kanneganti T.-D. (2010). Caspase-7: A Protease Involved in Apoptosis and Inflammation. Int. J. Biochem. Cell Biol..

[37] Mérino D., Lalaoui N., Morizot A., Schneider P., Solary E., Micheau O. (2006). Differential Inhibition of TRAIL-Mediated DR5-DISC Formation by Decoy Receptors 1 and 2. Mol. Cell. Biol..

[38] Chima M., Lebwohl M. (2018). TNF Inhibitors for Psoriasis. Semin. Cutan. Med. Surg..

[39] Koyani C.N., Windischhofer W., Rossmann C., Jin G., Kickmaier S., Heinzel F.R., Groschner K., Alavian-Ghavanini A., Sattler W., Malle E. (2014). 15-Deoxy-Δ12,14-PGJ2 Promotes Inflammation and Apoptosis in Cardiomyocytes via the DP2/MAPK/TNFα Axis. Int. J. Cardiol..

[40] Joo H.W., Kang Y.R., Kwack M.H., Sung Y.K. (2016). 15-Deoxy Prostaglandin J2, the Nonenzymatic Metabolite of Prostaglandin D2, Induces Apoptosis in Keratinocytes of Human Hair Follicles: A Possible Explanation for Prostaglandin D2-Mediated Inhibition of Hair Growth. Naunyn-Schmiedebergs Arch. Pharmacol..

[41] Lee S.J., Kim M.S., Park J.Y., Woo J.S., Kim Y.K. (2008). 15-Deoxy-Δ12,14-Prostaglandin J2 Induces Apoptosis via JNK-Mediated Mitochondrial Pathway in Osteoblastic Cells. Toxicology.

[42] Wójcik P., Biernacki M., Wroński A., Łuczaj W., Waeg G., Žarković N., Skrzydlewska E. (2019). Altered Lipid Metabolism in Blood Mononuclear Cells of Psoriatic Patients Indicates Differential Changes in Psoriasis Vulgaris and Psoriatic Arthritis. Int. J. Mol. Sci..

[43] Ambrożewicz E., Wójcik P., Wroński A., Łuczaj W., Jastrząb A., Žarković N., Skrzydlewska E. (2018). Pathophysiological Alterations of Redox Signaling and Endocannabinoid System in Granulocytes and Plasma of Psoriatic Patients. Cells.

[44] Lenhausen A.M., Wilkinson A.S., Lewis E.M., Dailey K.M., Scott A.J., Khan S., Wilkinson J.C. (2016). Apoptosis Inducing Factor Binding Protein PGAM5 Triggers Mitophagic Cell Death That Is Inhibited by the Ubiquitin Ligase Activity of X-Linked Inhibitor of Apoptosis. Biochemistry.

[45] Guo X., Sesaki H., Qi X. (2014). Drp1 Stabilizes P53 on the Mitochondria to Trigger Necrosis under Oxidative Stress Conditions in Vitro and in Vivo. Biochem. J..

[46] Liu A., Zhao W., Zhang B., Tu Y., Wang Q., Li J. (2020). Cimifugin Ameliorates Imiquimod-Induced Psoriasis by Inhibiting Oxidative Stress and Inflammation via NF-ΚB/MAPK Pathway. Biosci. Rep..

[47] Park J.S., Kang D.H., Lee D.H., Bae S.H. (2015). PF-4708671, a Specific Inhibitor of P70 Ribosomal S6 Kinase 1, Activates Nrf2 by Promoting P62-Dependent Autophagic Degradation of Keap1. Biochem. Biophys. Res. Commun..

[48] Dobrzyńska I., Szachowicz-Petelska B., Skrzydlewska E., Figaszewski Z.A. (2016). Effects of UVB Radiation on the Physicochemical Properties of Fibroblasts and Keratinocytes. J. Membr. Biol..

[49] Kim K.M., Im A.-R., Park S.K., Shin H.S., Chae S.-W. (2019). Protective Effects of Timosaponin AIII against UVB-Radiation Induced Inflammation and DNA Injury in Human Epidermal Keratinocytes. Biol. Pharm. Bull..

[50] Soonthornchai W., Tangtanatakul P., Meephansan J., Ruchusatsawat K., Reantragoon R., Hirankarn N., Wongpiyabovorn J. (2019). Down-Regulation of MiR-155 after Treatment with Narrow-Band UVB and Methotrexate Associates with Apoptosis of Keratinocytes in Psoriasis. Asian Pac. J. Allergy Immunol..

[51] Morita A. (2018). Current Developments in Phototherapy for Psoriasis. J. Dermatol..

[52] Leweke F.M., Piomelli D., Pahlisch F., Muhl D., Gerth C.W., Hoyer C., Klosterkötter J., Hellmich M., Koethe D. (2012). Cannabidiol Enhances Anandamide Signaling and Alleviates Psychotic Symptoms of Schizophrenia. Transl. Psychiatry.

[53] Chan J.Z., Duncan R.E. (2021). Regulatory Effects of Cannabidiol on Mitochondrial Functions: A Review. Cells.

[54] Wu Y., Zhao D., Zhuang J., Zhang F., Xu C. (2016). Caspase-8 and Caspase-9 Functioned Differently at Different Stages of the Cyclic Stretch-Induced Apoptosis in Human Periodontal Ligament Cells. PLoS ONE.

[55] Deshmukh J., Pofahl R., Haase I. (2017). Epidermal Rac1 Regulates the DNA Damage Response and Protects from UV-Light-Induced Keratinocyte Apoptosis and Skin Carcinogenesis. Cell Death Dis..

[56] Noh D., Choi J.G., Huh E., Oh M.S. (2018). Tectorigenin, a Flavonoid-Based Compound of Leopard Lily Rhizome, Attenuates UV-B-Induced Apoptosis and Collagen Degradation by Inhibiting Oxidative Stress in Human Keratinocytes. Nutrients.

[57] Rasheva V.I., Domingos P.M. (2009). Cellular Responses to Endoplasmic Reticulum Stress and Apoptosis. Apoptosis.

[58] Collier A.E., Wek R.C., Spandau D.F. (2015). Translational Repression Protects Human Keratinocytes from UVB-Induced Apoptosis through a Discordant EIF2 Kinase Stress Response. J. Investig. Dermatol..

[59] Banerjee A., Ahmed H., Yang P., Czinn S.J., Blanchard T.G. (2016). Endoplasmic Reticulum Stress and IRE-1 Signaling Cause Apoptosis in Colon Cancer Cells in Response to Andrographolide Treatment. Oncotarget.

[60] Petrovici A.R., Simionescu N., Sandu A.I., Paraschiv V., Silion M., Pinteala M. (2021). New Insights on Hemp Oil Enriched in Cannabidiol: Decarboxylation, Antioxidant Properties and In Vitro Anticancer Effect. Antioxidants.

[61] Niture S.K., Jaiswal A.K. (2013). Nrf2-Induced Antiapoptotic Bcl-XL Protein Enhances Cell Survival and Drug Resistance. Free Radic. Biol. Med..

[62] Murata K., Oyama M., Ogata M., Fujita N., Takahashi R. (2021). Oral Administration of Jumihaidokuto Inhibits UVB-Induced Skin Damage and Prostaglandin E2 Production in HR-1 Hairless Mice. J. Nat. Med..

[63] Coras R., Kavanaugh A., Boyd T., Huynh Q., Pedersen B., Armando A.M., Dahlberg-Wright S., Marsal S., Jain M., Paravar T. (2019). Pro- and Anti-Inflammatory Eicosanoids in Psoriatic Arthritis. Metabolomics.

[64] Zou S., Kumar U. (2018). Cannabinoid Receptors and the Endocannabinoid System: Signaling and Function in the Central Nervous System. Int. J. Mol. Sci..

[65] Zheng D., Bode A.M., Zhao Q., Cho Y.-Y., Zhu F., Ma W.-Y., Dong Z. (2008). The Cannabinoid Receptors Are Required for UV-Induced Inflammation and Skin Cancer Development. Cancer Res..

[66] Martínez-Martínez E., Martín-Ruiz A., Martín P., Calvo V., Provencio M., García J.M. (2016). CB2 Cannabinoid Receptor Activation Promotes Colon Cancer Progression via AKT/GSK3β Signaling Pathway. Oncotarget.

[67] Derkinderen P., Ledent C., Parmentier M., Girault J.A. (2001). Cannabinoids Activate P38 Mitogen-Activated Protein Kinases through CB1 Receptors in Hippocampus. J. Neurochem..

[68] Liu Z., Wang Y., Zhao H., Zheng Q., Xiao L., Zhao M. (2014). CB2 Receptor Activation Ameliorates the Proinflammatory Activity in Acute Lung Injury Induced by Paraquat. BioMed Res. Int..

[69] Ruhaak L.R., Felth J., Karlsson P.C., Rafter J.J., Verpoorte R., Bohlin L. (2011). Evaluation of the Cyclooxygenase Inhibiting Effects of Six Major Cannabinoids Isolated from Cannabis Sativa. Biol. Pharm. Bull..

[70] Black A.T., Gray J.P., Shakarjian M.P., Mishin V., Laskin D.L., Heck D.E., Laskin J.D. (2008). UVB Light Upregulates Prostaglandin Synthases and Prostaglandin Receptors in Mouse Keratinocytes. Toxicol. Appl. Pharmacol..

[71] Nagarkatti P., Pandey R., Rieder S.A., Hegde V.L., Nagarkatti M. (2009). Cannabinoids as Novel Anti-Inflammatory Drugs. Future Med. Chem..

[72] Sangiovanni E., Fumagalli M., Pacchetti B., Piazza S., Magnavacca A., Khalilpour S., Melzi G., Martinelli G., Dell’Agli M. (2019). *Cannabis Sativa* L. Extract and Cannabidiol Inhibit in Vitro Mediators of Skin Inflammation and Wound Injury. Phytother. Res..

[73] Sun Y., He L., Wang T., Hua W., Qin H., Wang J., Wang L., Gu W., Li T., Li N. (2020). Activation of P62-Keap1-Nrf2 Pathway Protects 6-Hydroxydopamine-Induced Ferroptosis in Dopaminergic Cells. Mol. Neurobiol..

[74] Casares L., García V., Garrido-Rodríguez M., Millán E., Collado J.A., García-Martín A., Peñarando J., Calzado M.A., de la Vega L., Muñoz E. (2020). Cannabidiol Induces Antioxidant Pathways in Keratinocytes by Targeting BACH1. Redox Biol..

[75] Ramer R., Heinemann K., Merkord J., Rohde H., Salamon A., Linnebacher M., Hinz B. (2013). COX-2 and PPAR-γ Confer Cannabidiol-Induced Apoptosis of Human Lung Cancer Cells. Mol. Cancer Ther..

[76] Pucci M., Rapino C., Di Francesco A., Dainese E., D’Addario C., Maccarrone M. (2013). Epigenetic Control of Skin Differentiation Genes by Phytocannabinoids. Br. J. Pharmacol..

[77] Rieder S.A., Chauhan A., Singh U., Nagarkatti M., Nagarkatti P. (2010). Cannabinoid-Induced Apoptosis in Immune Cells as a Pathway to Immunosuppression. Immunobiology.

[78] Wu H.-Y., Chu R.-M., Wang C.-C., Lee C.-Y., Lin S.-H., Jan T.-R. (2008). Cannabidiol-Induced Apoptosis in Primary Lymphocytes Is Associated with Oxidative Stress-Dependent Activation of Caspase-8. Toxicol. Appl. Pharmacol..

[79] Gęgotek A., Domingues P., Wroński A., Ambrożewicz E., Skrzydlewska E. (2019). The Proteomic Profile of Keratinocytes and Lymphocytes in Psoriatic Patients. Proteom. Clin. Appl..

[80] Behrens S., Grundmann-Kollmann M., Schiener R., Peter R.U., Kerscher M. (2000). Combination Phototherapy of Psoriasis with Narrow-Band UVB Irradiation and Topical Tazarotene Gel. J. Am. Acad. Dermatol..

[81] Fotakis G., Timbrell J.A. (2006). In Vitro Cytotoxicity Assays: Comparison of LDH, Neutral Red, MTT and Protein Assay in Hepatoma Cell Lines following Exposure to Cadmium Chloride. Toxicol. Lett..

[82] Jastrząb A., Gęgotek A., Skrzydlewska E. (2019). Cannabidiol Regulates the Expression of Keratinocyte Proteins Involved in the Inflammation Process through Transcriptional Regulation. Cells.

[83] Eissa S., Seada L.S. (1998). Quantitation of Bcl-2 Protein in Bladder Cancer Tissue by Enzyme Immunoassay: Comparison with Western Blot and Immunohistochemistry. Clin. Chem..

[84] Bradford M.M. (1976). A Rapid and Sensitive Method for the Quantitation of Microgram Quantities of Protein Utilizing the Principle of Protein-Dye Binding. Anal. Biochem..

[85] Gęgotek A., Domingues P., Wroński A., Wójcik P., Skrzydlewska E. (2018). Proteomic Plasma Profile of Psoriatic Patients. J. Pharm. Biomed. Anal..

