# Supplementary material for: Disease-Dependent Antiapoptotic Effects of Cannabidiol for Keratinocytes Observed upon UV Irradiation
Source: Int J Mol Sci. 2021 Sep 15;22(18):9956. doi: 10.3390/ijms22189956 (PMC8470797; doi:10.3390/ijms22189956)

Disease-dependent anti-apoptotic effects of cannabidiol for keratinocytes observed upon UV-irradiation

Piotr Wójcik, Agnieszka Gęgotek , Neven Žarković and Elżbieta Skrzydlewska

Supplementary

Western blots for parameters from Figure 1:

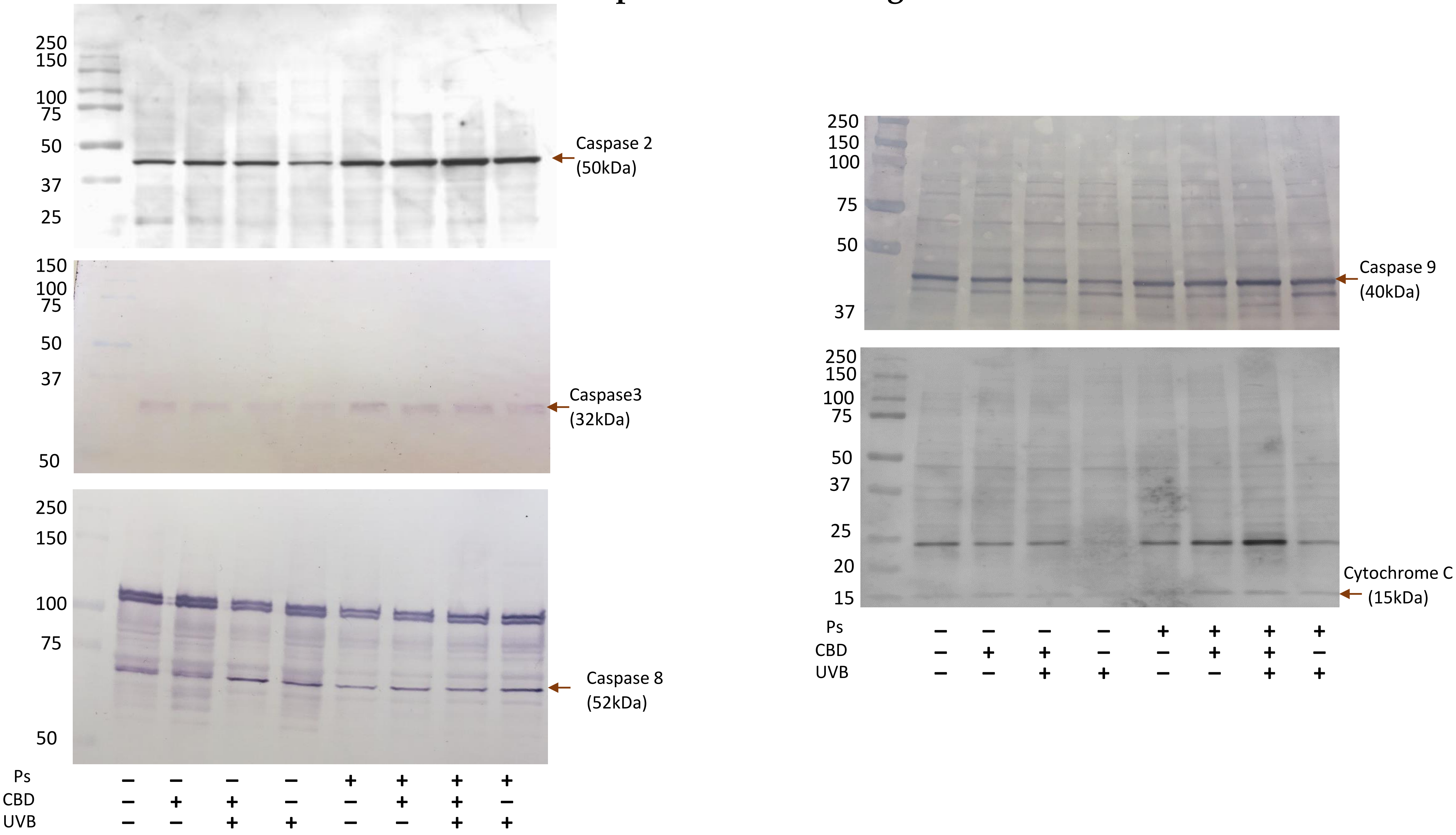

Western blots for parameters from Figure 2:

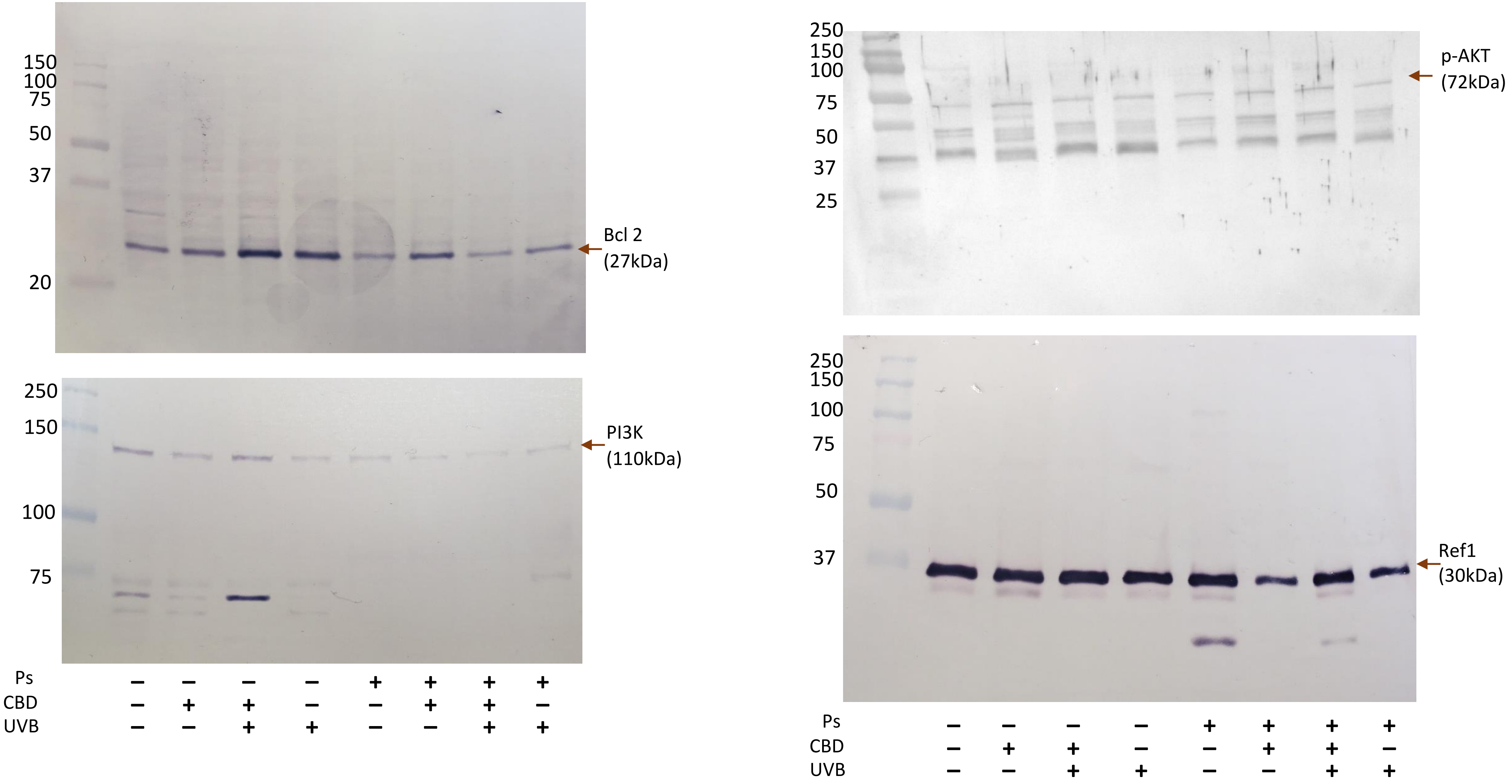

Western blots for parameters from Figure 3:

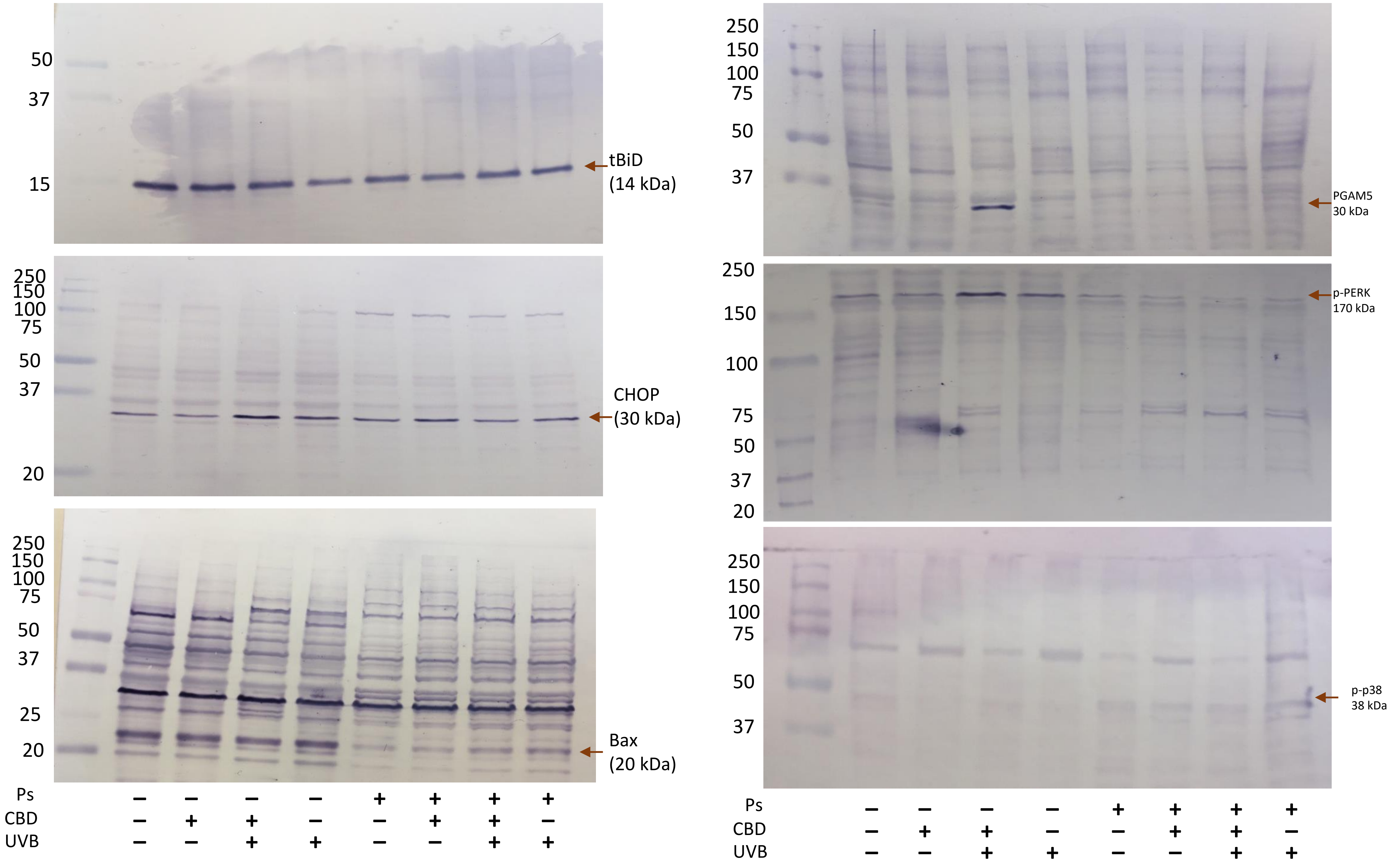

Western blots for parameters from Figure 3 (continued):

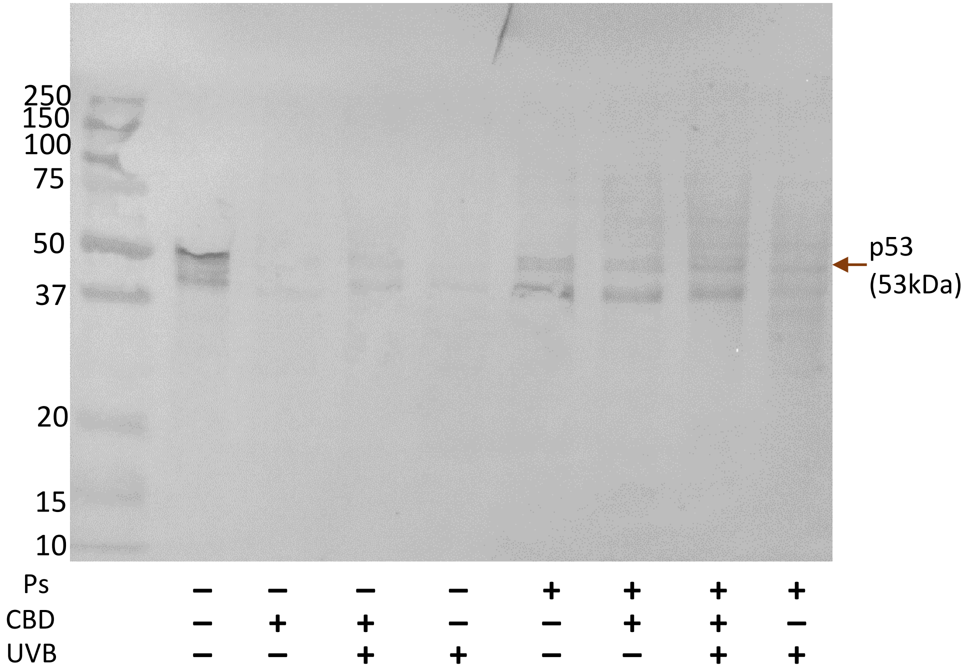

Western blots for parameters from Figure 4:

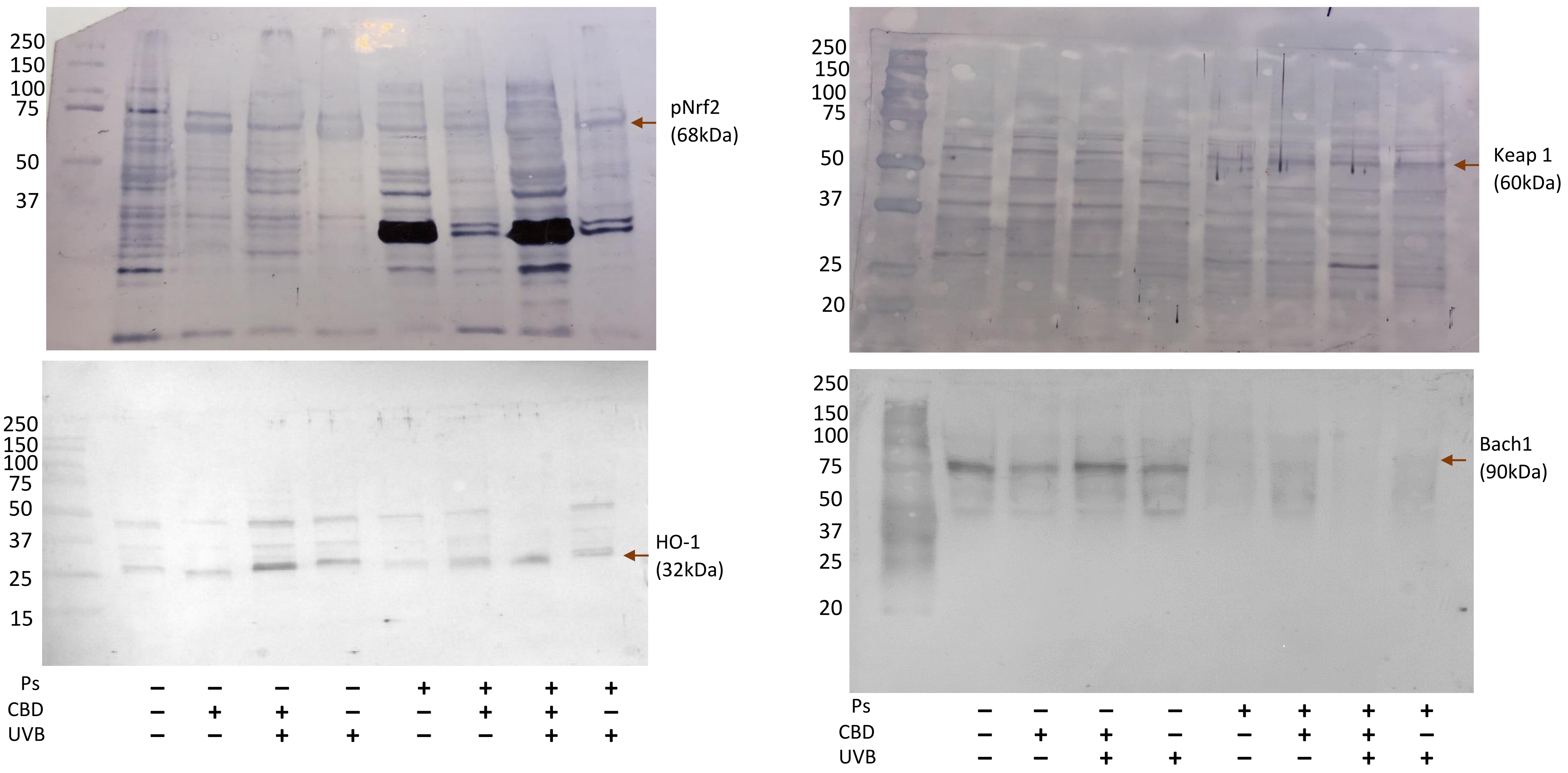

Supplement: Supplementary file 1 [file ijms-22-09956-s001.zip › Disease-dependent anti-apoptotic effects of cannabidiol for keratinocytes observed upon UV-irradiation.pdf]
