# Supplementary material for: Disease-Dependent Antiapoptotic Effects of Cannabidiol for Keratinocytes Observed upon UV Irradiation
Source: Int J Mol Sci. 2021 Sep 15;22(18):9956. doi: 10.3390/ijms22189956 (PMC8470797; doi:10.3390/ijms22189956)
Supplement: Supplementary file 1 [file ijms-22-09956-s001.zip › Supplementary.pdf]

Disease-dependent anti-apoptotic effects of cannabidiol for keratinocytes observed upon UV-irradiation

Piotr Wójcik[1], Agnieszka Gęgotek[1] , Neven Žarković[2] and Elżbieta Skrzydlewska[1,*]

Supplementary

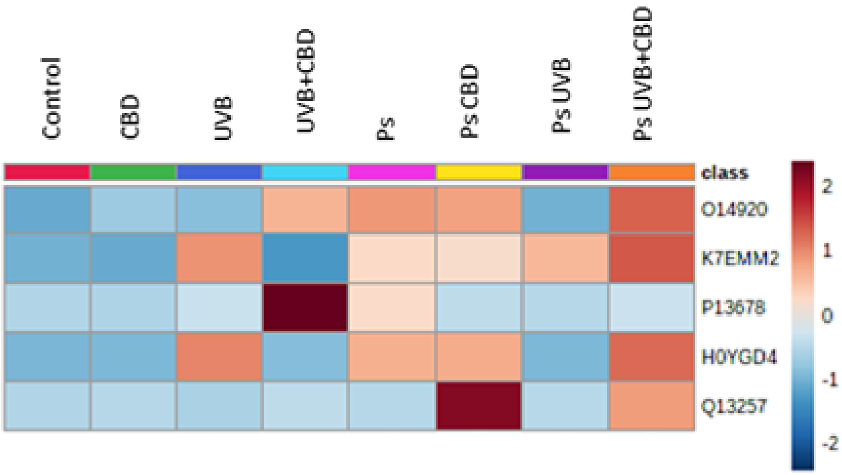

Figure S1.

Keap1 adducts in keratinocyte cell lines from heathy subjects (Control; n=5) and psoriatic patients (Ps; n=5) after irradiation with UVB (60 mJ/cm^2) and incubation with CBD (4µM). Heat map showing preliminary results of relative protein abundance of proteins making Keap1 complexes in keratinocytes from healthy subjects and psoriatic patients (Ps) after UVB exposure and incubation with CBD . Proteins IDs: O14920 - inhibitor of nuclear factor kappa-B kinase (IKKB).; K7EMM2 - ribosomal protein S6 kinase; P13678- protein kinase C; H0YGD4 - G1/S-specific cyclin D2; Q13257 - protein p62.

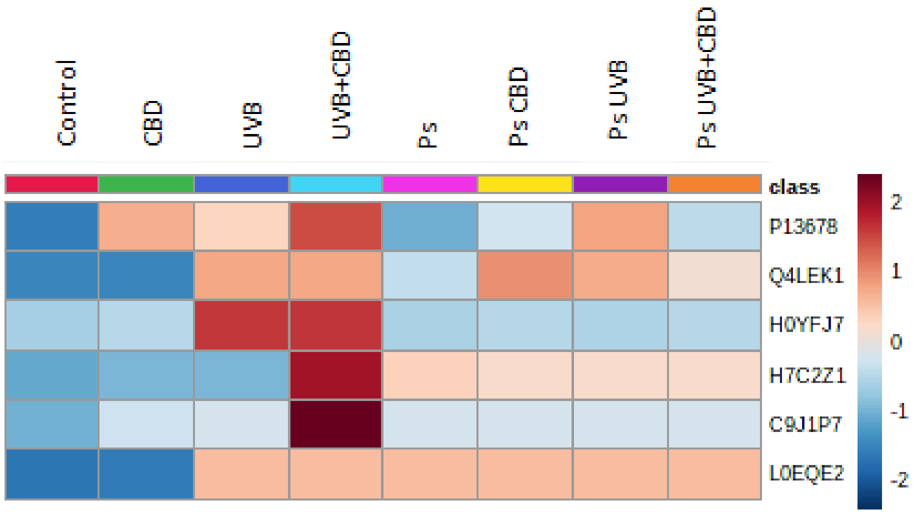

Figure S2.

PGAM5-protein adducts in keratinocyte cell lines from heathy subjects (Control; n=5) and psoriatic patients (Ps; n=5) after irradiation with UVB (60 mJ/cm^2) and incubation with CBD (4μM). Heatmap showing preliminary results of relative protein abundance of proteins making PGAM5 complexes in keratinocytes from healthy subjects and psoriatic patients (Ps) after UVB exposure and incubation with CBD . Proteins IDs: P13678 - protein kinase c; Q4LEK1 - prostanoid F receptor; H0YFJ7 - cell division cycle and apoptosis regulator protein 1; H7C2Z1 - apoptogenic protein 1 (mitochondrial); C9J1P7 - G-protein-coupled receptor 55 (GPRR55); L0EQE2 - p53 protein.
